# Developing Toxic Metal Environmental Justice Indices (TM-EJIs) for Arsenic, Cadmium, Lead, and Manganese Contamination in Private Drinking Wells in North Carolina

**DOI:** 10.3390/w14132088

**Published:** 2022-06-29

**Authors:** Noemi Gavino-Lopez, Lauren A. Eaves, Adam E. Enggasser, Rebecca C. Fry

**Affiliations:** 1 Department of Environmental Health Sciences and Engineering, Gillings School of Global Public Health, University of North Carolina at Chapel Hill, Chapel Hill, NC 27599, USA; 2 Institute for Environmental Health Solutions, Gillings School of Global Public Health, University of North Carolina at Chapel Hill, Chapel Hill, NC 27599, USA

**Keywords:** environmental justice, private wells, toxic metals

## Abstract

Toxic metal exposure via private drinking wells is an environmental health challenge in North Carolina (NC). Policies tainted by environmental racism shape who has access to public water supplies, with Black People, Indigenous People, and People of Color (BIPOC) often excluded from municipal services. Thus, toxic metal exposure via private wells is an environmental justice (EJ) issue, and it is under-studied in NC. In this study, we developed four Toxic Metal Environmental Justice Indices (TM-EJIs) for inorganic arsenic (iAs), cadmium (Cd), lead (Pb), and manganese (Mn) to quantitatively identify areas of environmental injustice in NC. TM-EJIs were calculated at the census tract level (n = 2038) as the product of the following: (1) number of well water tests with concentrations exceeding national standards, (2) percentage of the low-income and minority population, and (3) population density. Mn had the greatest proportion (25.17%) of positive TM-EJIs, which are indicative of socioeconomically disadvantaged groups exposed to toxic metals. Positive TM-EJIs, particularly for Pb and Mn, were primarily located in eastern NC. These results highlight several new counties of concern and can be used by public health professionals and state environmental agencies to prioritize remediation efforts and efforts to reduce environmental injustices.

## 1. Introduction

Unlike public drinking water systems, which are protected by the Environmental Protection Agency’s (EPA) Safe Drinking Water Act, private wells are not federally regulated in the United States [1]. Although individual states have the authority to establish policies governing private wells, the extent of these policies vary and the policies are often focused on legal requirements for drilling and constructing wells [2]. The lack of biological and chemical contaminant standards for private wells at the state and federal level leaves approximately 45 million American users at a higher risk of exposure to waterborne contaminants [3]. Known groundwater contaminants in the U.S. include toxic metals/metalloids such as inorganic arsenic (iAs), cadmium (Cd), lead (Pb), and manganese (Mn), all of which are on the ATSDR’s Substance Priority List [4]. These metals/metalloids are known to impact the immune, cardiovascular, nervous, and renal systems, leading to widespread health effects, including cancer [5-8]. For example, long-term exposure to iAs-contaminated drinking water is associated with cancers of the skin, bladder, and lung [9]. Other health effects of iAs exposure include diabetes, pulmonary disease, and cardiovascular disease [9]. Exposure to high levels of Cd is associated with cancers of the lung and prostate [10]. Furthermore, Cd’s ability to accumulate in various human organs can result in kidney, liver, bone, and blood damage [11]. Pb can lead to decreased kidney function alongside reproductive problems, cardiovascular effects, and adverse neurological outcomes [12]. Lastly, exposure to Mn-contaminated drinking water can cause neurological effects, affecting memory, attention, and motor skills [13]. Evidence also suggests that prenatal exposure to these contaminants is associated with adverse birth and developmental outcomes such as low birth weight, preterm birth, spontaneous abortion, and neurodevelopmental impairment [14,15].

As a result of the adverse health effects of these contaminants, EPA has established maximum contaminant levels (MCLs) for iAs and Cd, a secondary maximum contaminant level (SMCL) for Mn, and an action plan level for Pb. An MCL is the highest level of a contaminant that is allowed in public drinking water systems and the limit is generally enforceable by public health authorities [16]. An SMCL is a non-mandatory water quality standard for public water systems established for aesthetic reasons, including taste, odor, and color; contaminants at or below the SMCL do not present a risk to human health [17]. An action level is not a standard, but a measure of the effectiveness of corrosion control treatment in public drinking water systems. If 10% of household samples contain a contaminant above the action level, the public water system must inform the public and perform service line replacements [18]. The MCLs for iAs and Cd are 10 ppb and 5 ppb, respectively [16]. The SCML for Mn is 50 ppb, and the action level for Pb is 15 ppb [17,18]. Herein, for simplicity, these metal concentration standards and guidelines will be referred to as maximum contaminant levels (MCLs).

A household’s source of drinking water is primarily determined by the availability of a municipal water source. In NC and elsewhere, racial minorities and low-income individuals are found to be underserved with respect to municipal water and sewer services [19]. This is partially a result of institutional racism ingrained in municipal zoning practices known as “underbounding,” in which the water and sewer service boundaries have historically been intentionally underdrawn in the U.S. South to exclude minorities [20,21]. As a result, these communities are forced to rely on private wells, despite the proximity of a municipal water system; this places them at a higher risk for chemical exposure via water, with the corresponding health risks. Concurrently, low-socioeconomic status is a barrier that prevents private well owners from achieving better water quality due to the costs of testing and remediation efforts [22]. In North Carolina, the cost of testing for inorganic contaminants in private wells varies by the local health department, but can range from USD 35 to USD 250 [23]. Treatment options for contaminated wells include kitchen tap filters or under-sink filters, but these present additional costs ranging from USD 200 to USD 400 [24]. Therefore, exposure to toxic metals via private well contamination is an imminent public health concern, particularly among low-income and minority populations, who often experience disproportionate health effects a result of adverse environmental exposures [20].

These concerns regarding disproportionate exposure to contaminated well water are connected with the larger environmental justice (EJ) movement to eliminate the differential exposure of any population to environmental hazards [25]. EJ initiatives are gaining momentum and have been a focus of President Biden’s Executive Order 14,008 and Justice 40 initiative calling for 40% of federal environmental investment funding to target disadvantaged communities across the country [26]. As a result of this push, two federal tools have been created to visualize the intersection of exposures and demographic risks, including EPA’s EJSCREEN tool and a Climate and Economic Justice Screening Tool to highlight areas of concern [27,28]. Similarly, California and Maryland have created related databases and tools to advance EJ initiatives [29,30]. However, there are limited methods by which to explore EJ issues resulting from toxic metal exposures via private well contamination in North Carolina (NC).

NC is an ideal location to examine the intersection of the aforementioned factors. Of all residents, 24%, or 2.4 million, utilize private wells for drinking water, making the state’s residents the fifth largest population in the country relying on private water sources [31]. We recently documented that thousands of these wells are contaminated with toxic metals exceeding the EPA’s recommended limits, with comprehensive data publicly available in the NCWELL database [32]. Furthermore, these toxic metal concentrations in NC wells have been linked to biomarkers of exposure, such as elevated blood lead levels, and adverse health effects, including neurodevelopmental impairment, hearing loss, and heart defects [14,15,24,33]. Together, these data suggest that disadvantaged communities may be at risk for increased exposure to toxic metals via drinking water contamination.

The goal of the present study was to create indices for understanding the intersection of demographics and private well contamination of toxic metals in NC. To perform this, we adapted the EPA’s existing EJ Index format for application to private well water contamination and constructed EJIs for four toxic metals/metalloids, namely, iAs, Cd, Mn, and Pb, at a census tract level [27]. The resulting indices identified areas of potential environmental injustice stemming from private well water contamination, highlighting counties for further investigation [27].

## 2. Materials and Methods

### 2.1. Overview ofTM-EJI Construction

**Figure 1.**
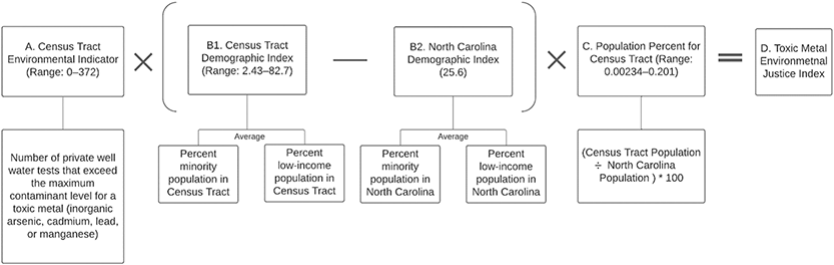
Mathematical formula for TM-EJIs, a product of an environmental indicator, a demographic index, and a census tract’s population (* indicates a multiplication factor).

We calculated EJIs at a census tract level for iAs, Cd, Mn, and Pb–metals that are known to contaminate NC private wells [34]. Figure 1 outlines the construction of EJIs, which are calculated for each metal in each census tract by multiplying an environmental indicator (EI), a demographic index (DI), and a population variable.

### 2.2. Environmental Indicator Calculation

To go into greater detail, the environmental indicator was the number of well water tests that exceeded the maximum contaminant level for iAs (10 ppb), Cd (5 ppb), Mn (50 ppb), and Pb (15 ppb) in each tract. Data were obtained from the NCWELL database, which is composed of NC private well water tests collected from 1998 to 2019. Private well water samples were collected by local public health department officials, or upon request to the owner, and then analyzed at the NCDHHS Division of Public Health State Laboratory of Public Health. The database contains geocoded well water tests for iAs, Cd, Mn, and Pb that have been summarized for each census tract. Further details about the collection of private well water tests, analysis, and construction of this database are provided elsewhere [32]. For the purposes of this study, we utilized the number of samples in a tract that were at or above the EPA limit as the environmental indicator. The EPA limit used for each of these metals was: 10 ppb for iAs, 5 ppb for Cd, 50 ppb for Mn, and 15 ppb for Pb. These data were summarized as the number of private well tests that exceeded the EPA MCLs for each census tract. This portion of the TM-EJI formula contributes to the magnitude, but not to the direction, of EJIs.

### 2.3. Demographic Index Calculation

The DI was defined as the percentage of people in a tract’s population who were low-income or racial minorities relative to the state’s average percentage of low-income and racial minority populations. This formula is modeled upon the EPA’s EJ Index formula. Specifically, we calculated an NC DI by integrating the statewide percentage of the low-income population and the percentage of the minority population and averaging across the two percentages. Then, to calculate each census tract’s DI, we integrated data from the percentage of the low-income population and the percentage of the minority population for each census tract and, again, averaged across the two percentages. For final census tract DI, we calculated the difference between census tract DI and NC DI. Data used to calculate the DI’s were downloaded from the American Communities Survey 2019 using the tidycensus package in R. This portion of the TM-EJI formula determines the difference between the state average and census tract demographics, which may result in a positive or negative EJI.

### 2.4. Population Percent Calculation

Lastly, the percentage value a census tract contributes to the total NC population is also included in the TM-EJI formula. This variable is determined by dividing the census tract population by the NC population and then converting the result into a percentage. The percent a census tract contributes to the total NC population was used instead of a population count to prevent the magnitude of TM-EJIs being driven primarily by the population variable. By relying on population percentages, the magnitude of TM-EJIs is determined primarily by the environmental indicator and DI. Population data for each census tract were obtained from the 2019 ACS; the NC total population was determined by adding up the individual populations from the census tracts used in this dataset.

### 2.5. Data Analysis

Overall, there are *n* = 2195 census tracts in NC. For these analyses, *n* = 150 census tracts were removed from this analysis due to a lack of well water tests data, and *n* = 7 were removed due to missing demographic data. In total, *n* = 2038 NC census tracts had the necessary demographic and environmental data for calculating their corresponding EJIs. All data analysis was conducted in Excel (v16.54) and Python (v3.8.8).

A singular EJI, for a given census tract and metal, is the product of the three aforementioned items, and it may be positive or negative based on the demographic variables in the formula.

In order to determine geographic locations where low-income and minority populations are more susceptible to metal exposure via private well contamination in NC, the number of census tracts with positive EJIs was identified for each metal. To visualize the location of census tracts with positive EJIs, county-level maps were generated with QGIS (v3.16.12-Hanover). Furthermore, counties were ranked according to the percentage of census tracts that had positive EJIs for each metal to identify locations that require immediate public health attention.

## 3. Results

### 3.1. TM-EJIs Determined

**Figure 2.**
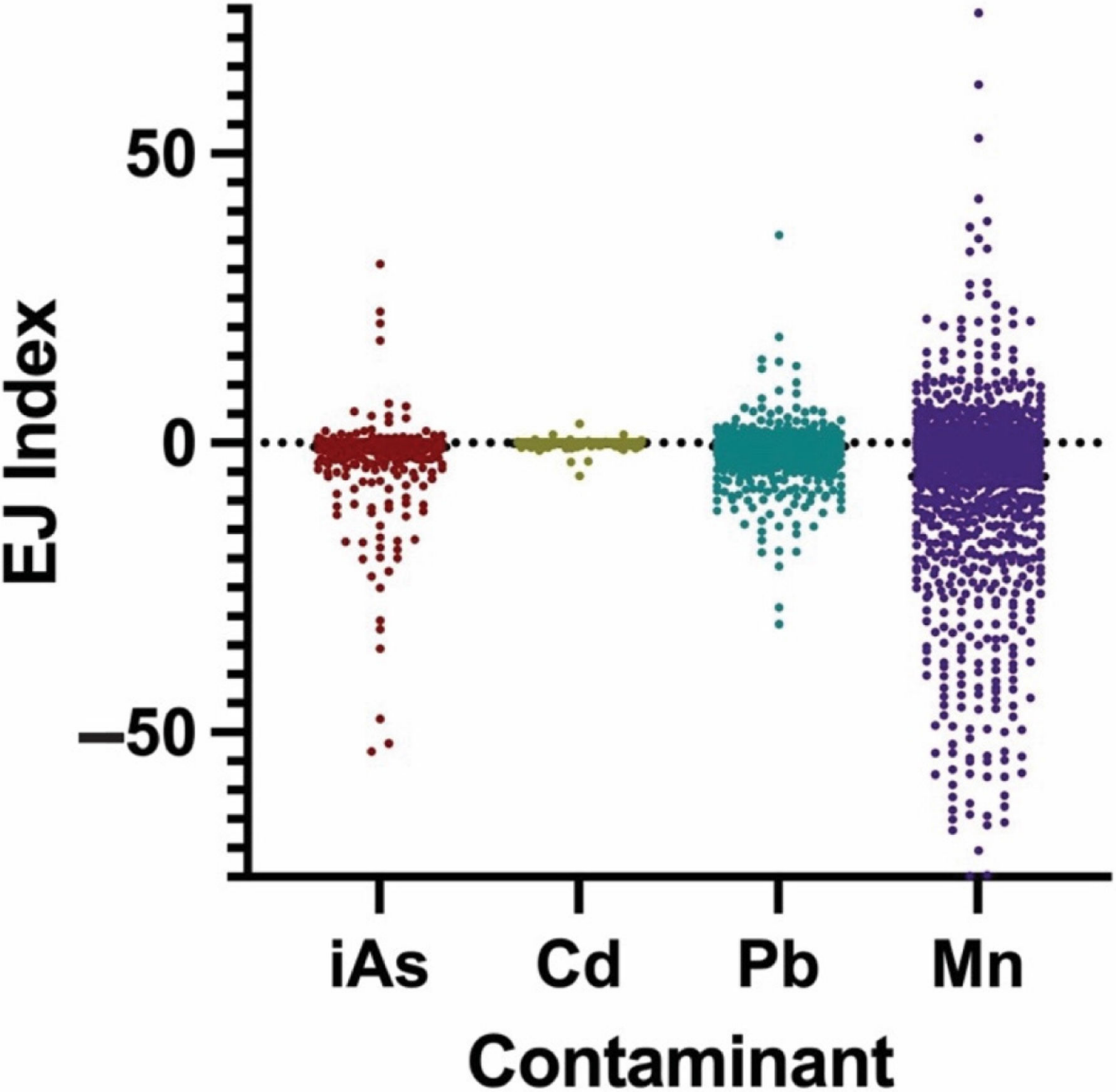
Distribution of EJIs for the four contaminants of interest: inorganic arsenic (iAs), cadmium (Cd), lead (Pb), and manganese (Mn). Positive EJIs represent contamination in disadvantaged areas.

A total of *n* = 8152 TM-EJIs were determined using the strategy depicted in Figure 1. Specifically, each NC census tract with available data (*n* = 2038) has four TM-EJI values: one for each metal of interest (iAs, Cd, Pb, and Mn). The raw values of *n* = 8152 TM-EJIs determined in this study are found in Table 1. For ease of interpretation, trends and patterns based on the number of EJIs that are positive, negative, or zero are reported for each metal (Table 1). A positive EJI occurs when the average population of low-income and minority individuals for a census tract is higher than the NC average. Census tracts with negative EJIs have a local DI below NC’s average or a lower proportion of low-income and minority residents than does the state’s average. The incorporation of positive and negative values in the EJI is useful for identifying census tracts that have higher or lower numbers of low-income and minority individuals susceptible to metal exposure via private wells than the expected amount based on NC’s demographics. An EJI equal to zero occurred in census tracts where no private well tests were reported above the standard for the corresponding metal. As presented in Table 1, Mn had the highest number of positive EJIs, followed by Pb, iAs, and finally Cd. It is important to note that the number of positive EJIs is driven by the number of tests in the NCWELL Database that exceeded the MCL for each metal. Furthermore, Figure 2. presents the distribution of positive, zero, and negative TM-EJIs in comparison to one another.

### 3.2. Positive TM-EJIs Identify Counties of Concern for iAs, Cd, Pb, and Mn Private Well Contamination among Low-Income and Minority Populations

**Figure 3.**
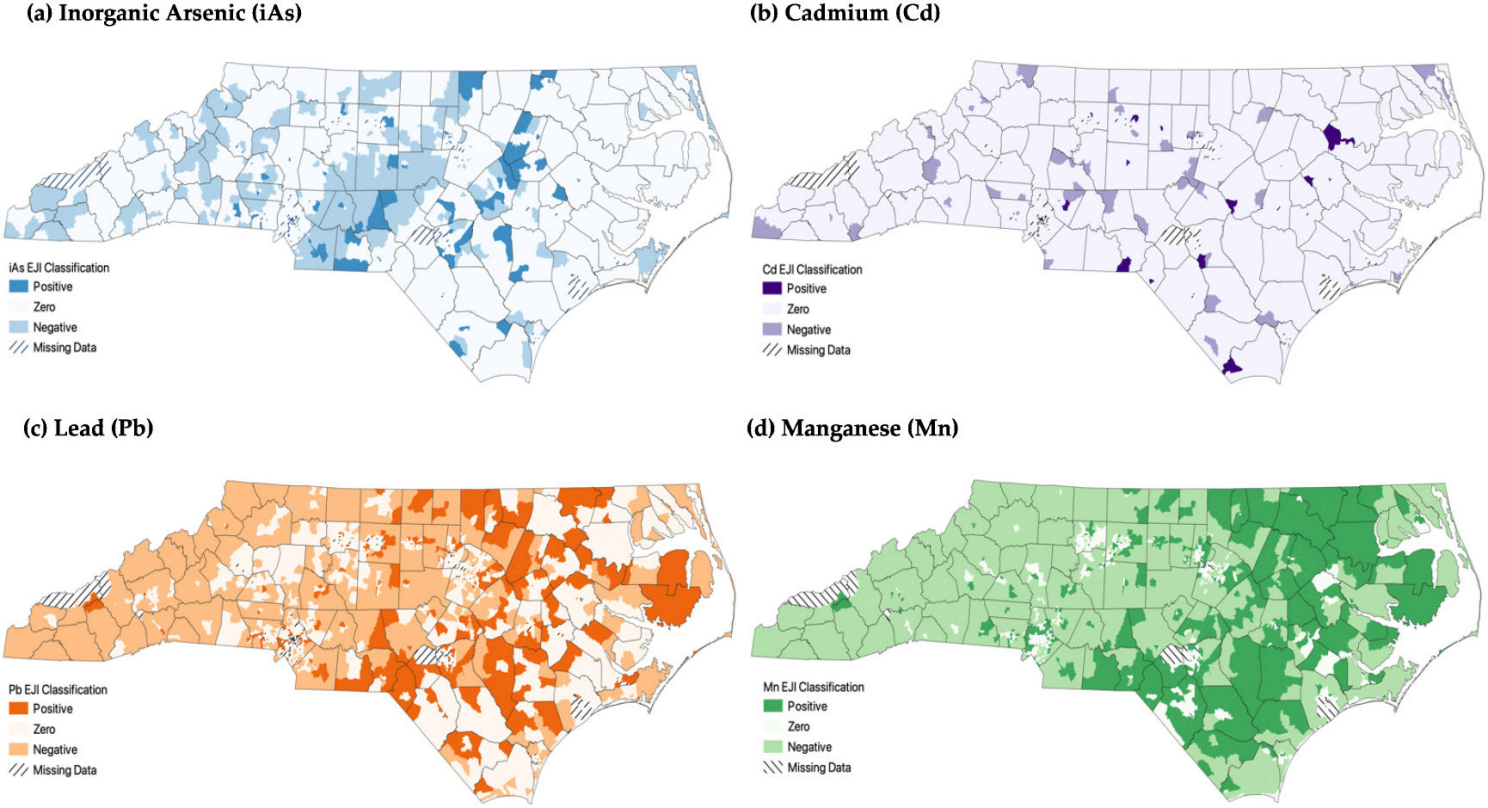
A map of North Carolina representing locations of (**a**) inorganic arsenic, (**b**) cadmium, (**c**) lead, and (**d**) manganese EJ Indices at the census tract level within counties. Dark colors indicate metal contamination in disadvantaged (low income/large minority population) census tracts, light areas indicate metal contamination in less disadvantaged (high income/small minority population) census tracts, and white areas indicate no contamination.

The locations of positive, negative, and zero TM-EJIs are represented by county-level maps and color categorization (Figure 3). For all four of the metals, positive TM-EJIs are generally found in the eastern portion of the state. This pattern is particularly prominent in maps for Pb and Mn. Positive EJIs were also observed in peri-urban areas (transition zones from rural to urban locations). For example, Guilford County has a prominent ring of positive EJIs for Pb and Mn and less noticeably for iAs and Cd.

### 3.3. Communities of Concern

**Figure 4.**
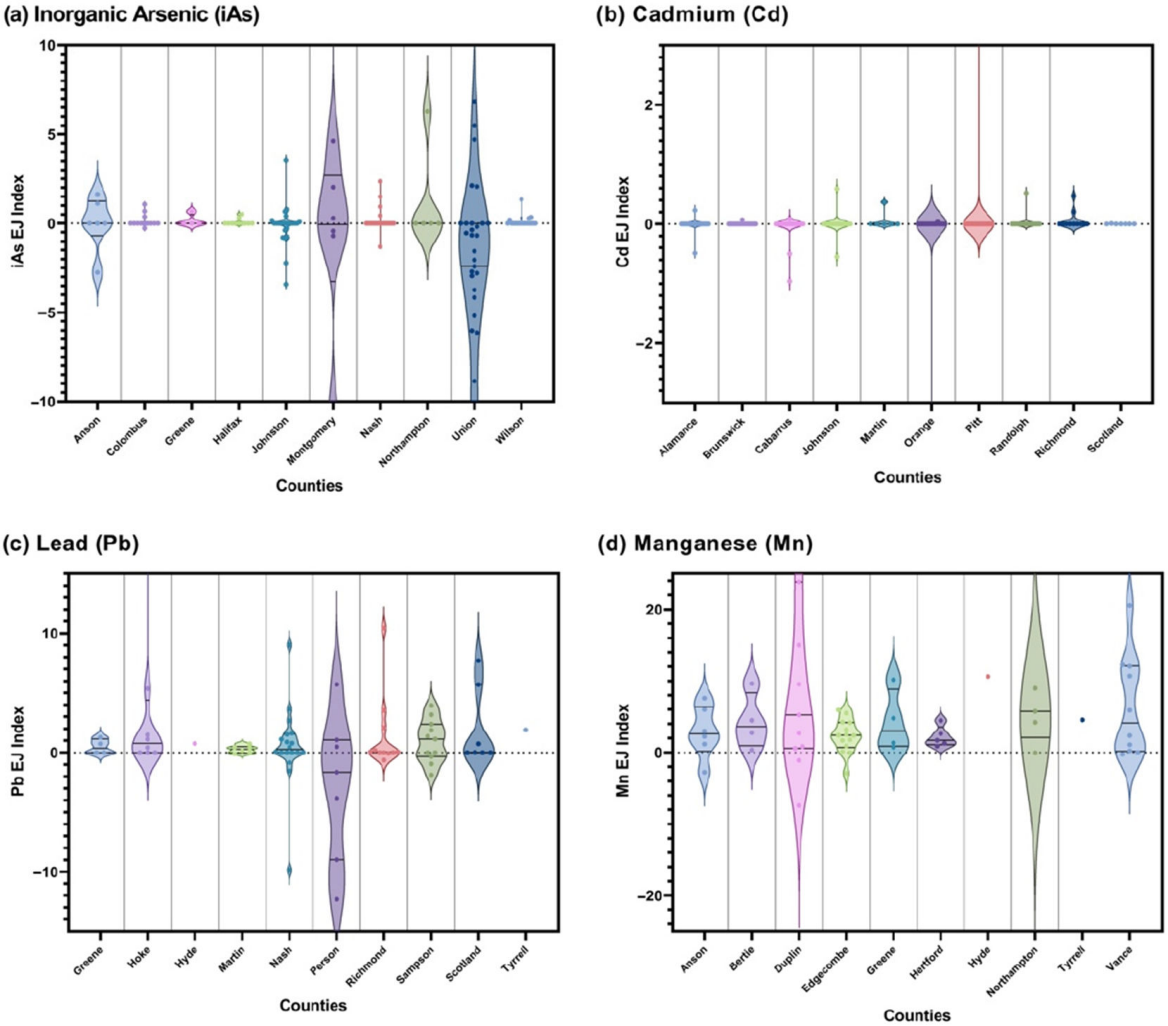
Counties with the highest EJIs for (**a**) inorganic arsenic, (**b**) cadmium, (**c**) lead, and (**d**) manganese contamination of private wells. Positive EJIs represent contamination in disadvantaged areas.

For iAs, the top ten counties of concern according to the percentage of positive EJIs are Montgomery, Anson, Nash, Greene, Johnston, Columbus, Wilson, Northampton, Union, and Halifax (Figure 4, Table 2). The county with the highest percentage of positive iAs EJIs is Montgomery at 50%. For Cd, the top ten counties according to the percentage of positive EJIs are Richmond, Martin, Scotland, Johnston, Orange, Randolph, Pitt, Brunswick, Alamance, and Cabarrus (Figure 4, Table 2). The percentage of positive Cd EJIs, among these counties, are all below 20%. For Pb, the top ten counties according to the percentage of positive EJIs are Hyde, Tyrrell, Martin, Hoke, Nash, Scotland, Sampson, Richmond, Greene, and Person (Figure 4, Table 2). Nine out of the top ten counties have a percentage of positive Mn EJIs at or above 50%. For Mn, the top ten counties according to the percentage of positive EJIs are: Hertford, Bertie, Green, Hyde, Tyrell, Edgecombe, Anson, Duplin, Vance, and Northampton (Figure 4, Table 2). These 10 counties all had a percentage of positive Mn EJIs at or above 80%.

## 4. Discussion

Exposure to toxic metals such as iAs, Cd, Mn, and Pb via private wells in NC is a comprehensively documented public health concern [32]. In addition, municipal underbounding has resulted in minority communities’ being intentionally excluded from municipal drinking water, leaving some demographics disproportionately forced to rely upon private wells [20]. Despite these known concerns, there is no quantitative tool that assesses environmental justice concerns as they relate to well water contamination in NC. This study fills this gap by developing four TM-EJIs at a census tract level for iAs, Cd, Mn, and Pb. TM-EJIs summarize how a single environmental factor (a metal in drinking water) and demographics intersect in a particular location, revealing disadvantaged communities that are disproportionately exposed to contaminated drinking water [27]. There are three major scientific contributions of the present study. First, we developed a method for identifying areas with potential environmental injustices stemming from private well water contamination, providing a model that can be used in other states as well. Second, we observed statewide trends in metal contamination that highlight environmental justice concerns in the eastern part of the state. Lastly, we identified specific counties of concern across the state, some of which not previously discussed in the literature. These findings will help target state and federal public health efforts and provide a model for other states investigating well-related environmental injustices.

At the national level, EPA has developed EJIs for 12 different environmental factors, including air pollution, lead paint, and proximity to hazardous waste [27]. These indices consider both the presence of environmental contaminants and population demographics to create a quantitative tool to predict environmental injustice occurrence. Similarly to the EPA’s EJScreen, the White House Council on Environmental Quality (CEQ) has recently released an initial version of a national tool that identifies census tracts with both elevated environmental/climate indicators and socioeconomic indicators [35]. At the state level, California and Maryland have created similar environmental justice screening tools to investigate environmental injustices in their states, tools that have since been adopted by state agencies to identify disadvantaged communities and inform funding [36]. However, there is no precedent for a methodology to create an EJI for toxic metal contamination of private well water. We chose to build our TM-EJIs upon the existing EPA EJScreen tool, substituting other environmental factors for the presence of metal concentrations exceeding EPA standards in well water samples. The EPA’s well-established EJScreen methodology was chosen as a template to provide consistency between platforms and research. The four metals examined (iAs, Cd, Pb, and Mn) are a significant concern in NC, as they have been observed to exceed EPA standards, and exposure to these metals is associated with adverse health effects such as birth defects, lower birthweight, and cancer [14,32]. The TM-EJIs determined in this study provide an innovative tool to identify locations in NC where susceptible populations (low-income and minority individuals) are at risk of exposure to metals via private well contamination.

Moreover, these TM-EJIs can be used to identify statewide trends or specific counties of concern. When examining NC as a whole, almost all positive EJIs were found in the eastern part of the state in the coastal plain region; very few positive indices were found west of the fall line. In particular, this trend was observed for Pb and Mn. This pattern was expected, as many disadvantaged communities are found in the eastern part of the state. TM-EJIs can only be positive if an area is disproportionately disadvantaged, so for metals contaminating wells across the state, the map of positive TM-EJIs closely resembles a map of disadvantaged areas. Comparing the four metals, Mn had the greatest number of positive EJIs and the highest positive EJIs, presenting a risk for environmental injustices in NC. Similarly, Pb had a significant proportion of positive EJIs, but a smaller proportion and magnitude than did Mn. In contrast, cadmium had few non-zero EJIs, but this may be due to the limited number of private well tests that analyzed Cd levels and fewer tests that measured Cd above the limit of reporting in the NC Well Database. iAs had mostly negative EJIs, but the 51 positive iAs EJIs present an environmental injustice in corresponding locations. While Mn had the largest number of positive EJIs and may appear as the number one metal of concern, it is important to note that Mn is an essential nutrient required in low doses for development, metabolism, and the antioxidant system [37]. Nevertheless, excessive exposure to Mn can adversely affect the nervous system [8]. In comparison, there are no safe levels of iAs, Cd and Pb, which are all uniformly toxic. Therefore, the positive EJIs for iAs, Cd, and Pb, although fewer than Mn, are still concerning.

TM-EJIs can also be used in the identification of counties with the highest percentage of positive EJIs for multiple metals, thereby flagging these counties for increased well water support programs and intervention. These counties include Anson, Greene, Hyde, Johnston, Nash, Northampton, Martin, Scotland, and Tyrell. Several of these counties have been noted before as counties of concern for toxic metals exposure, including Anson [32,38]. However, several of these counties, including Greene, Hyde, Northampton, and Martin, are newly highlighted here, as they represent the intersection of high metals and high socioeconomic vulnerability. Importantly, evaluating areas only for toxic metal exposure or only socioeconomic vulnerability masks environmental justice concerns. By ranking highly for multiple metals, counties may be identified as locations of concern for chemical co-occurrence that require immediate remediation efforts to prevent multi-metal exposure via private wells among low-income and minority populations. A second consideration is the identification of counties with large percentages of positive EJIs for a single metal. Notably, in the context of Pb exposure, several counties including Hyde, Tyrrell, Martin, Hoke, Nash, Scotland, Sampson, Richmond, and Greene had TM-EJIs ≥ 50%. Therefore, it is important to address Pb contamination, which presents health risks at any level, specifically among these counties, to reduce metal exposure among low income and minority individuals. Importantly, Pb may be removed from drinking water with a certified point-of-use filter (POU), a filtration device that is mounted onto the faucet or under the sink for water treatment. POU-activated carbon block filters reduce high concentrations of lead in drinking water [39]. To ensure the effectiveness of the filter, it must be NSF/ANSI 53 certified, meaning that it is capable of reducing contaminants with known health effects, including lead [40].

While TM-EJIs are an innovative tool for identifying locations where susceptible populations (low-income and minority individuals) are at risk of well water contamination, there are limitations to this study. First, the 2019 American Community Survey demographic estimates were used instead of census data. This demographic data source was prioritized due to its recency; at the time of analysis, the most recent available census was from 2010. However, the American Community Survey does not provide a full census of all households and may, therefore, introduce uncertainty into the EJIs. In the future, census data could be incorporated into the analysis. Another limitation is that the data provided here are at the census tract level, as opposed to block group level, providing decreased geographic resolution. Finally, the mapping approach used here prioritized the sign (positive/negative) of EJI rather than the magnitude of the value, displaying only one dimension of data.

In summary, by developing TM-EJIs for toxic metal contamination of private wells in NC, environmental justice efforts for providing equal protection of all communities against environmental hazards can be advanced. These EJIs may be used to identify locations that require immediate attention and regulatory efforts by public health officials and policymakers in order to reduce the environmental health burden of metal contaminated wells among disproportionality affected communities. Further research is necessary in order to reduce the uncertainty associated with the EJIs determined in this study.

## Figures and Tables

**Table 1.** Descriptive statistics for each of the four TM-EJIs generated.

Metal	Total	Negative	Zero	Positive	Maximum	Minimum	Mean
Inorganic Arsenic	2038	260	1707	71	30.9	−273	−0.693
Cadmium	2038	51	1973	14	3.26	−5.69	−0.0145
Lead	2038	637	1138	263	35.9	−31.3	−0.533
Manganese	2038	1012	513	513	80.2	−355	−5.82

**Table 2.** Counties with the ten highest percentages of positive TM-EJIs.

County	Census Tracts with Positive EJIs	Total Number of EJIs	Percentage of Positive EJIs
**iAS**			
Montgomery	3	6	50
Anson	2	6	33.33
Nash	5	18	27.78
Greene	1	4	25
Johnston	6	25	24
Columbus	3	13	23.08
Wilson	4	18	22.2
Northampton	1	5	20
Union	8	41	19.51
Halifax	2	11	18.18
**Cd**			
Richmond	2	11	18.18
Martin	1	6	16.67
Scotland	1	7	14.29
Johnston	1	25	4
Orange	1	27	3.7
Randolph	1	28	3.57
Pitt	1	30	3.33
Brunswick	1	32	3.125
Alamance	1	36	2.78
Cabarrus	1	36	2.78
**Pb**			
Hyde	1	1	100
Tyrrell	1	1	100
Martin	4	6	66.67
Hoke	5	8	62.5
Nash	11	18	61.11
Scotland	4	7	57.14
Sampson	6	11	54.55
Richmond	6	11	54.55
Greene	2	4	50
Person	3	7	42.86
**Mn**			
Hertford	5	5	100
Bertie	4	4	100
Greene	4	4	100
Hyde	1	1	100
Tyrrell	1	1	100
Edgecombe	12	14	85.71
Anson	5	6	83.33
Duplin	9	11	81.82
Vance	8	10	80
Northampton	4	5	80

## Data Availability

The NCWELL database is publicly available on the UNC Superfund Research Program UNC Dataverse, https://doi.org/10.15139/S3/BDQG9O (accessed on 1 October 2021) (Eaves et al., 2021).
